# Design of a novel dual-polarized microwave sensor for human bone fracture detection using reactive impedance surfaces

**DOI:** 10.1038/s41598-023-38039-3

**Published:** 2023-07-04

**Authors:** Aslan Nouri Moqadam, Robab Kazemi

**Affiliations:** Faculty of Electrical and Computer Engineering, University of Tabriz, Tabriz, 5166616471 Iran

**Keywords:** Biomedical engineering, Electrical and electronic engineering

## Abstract

This paper presents a novel miniaturized dual-polarized transceiver sensor system for detecting fractures in human bone tissues. The system features a patch antenna and a Reactive Impedance Surface (RIS) layer that reduces its size by 30% compared to conventional designs, resulting in enhanced fracture detection accuracy. Additionally, the system includes a dielectric plano-concave lens that adapts to the human body and improves impedance matching for optimal performance. The lens contains via holes filled with a lossy dielectric material similar to human fat tissue, which concentrates electromagnetic (EM) power and increases penetration depth for more effective crack detection. To detect fractures, two identical sensors are placed opposite each other on the tissue and moved simultaneously. The amount of EM power collected by the receiver sensor is measured using S-parameters; the transmission coefficient (S_21_) phases and contrast between the crack and surrounding tissue are used to construct images of fractured bones. Full-wave simulations and experimental measurements on a semi-solid human arm mimicking phantom demonstrate the proposed dual-polarized sensor's ability to detect the location and orientation of narrow cracks in the millimeter range. The system exhibits reliable performance across different human bodies.

## Introduction

Microwave imaging and the use of microwaves in Non-Destructive Testing and Evaluation (NDT&E) have advanced significantly, making it a reliable alternative to traditional medical imaging methods in medical applications, such as tumor detection in various tissues. The accuracy and low cost of high-quality microwave sensors in defect detection make non-destructive testing widely used in industrial and medical applications. Researchers are increasingly interested in non-invasive medical diagnosis, and microwave imaging is a suitable option due to its safety and precision. While X-ray imaging and CT scans are commonly used techniques for medical imaging, their ionizing characteristics can be harmful with frequent use, particularly for elderly patients. For certain applications such as bone fracture detection and monitoring healing progression, frequent imaging is necessary, which limits the use of CT or X-ray scans.

Non-invasive microwave imaging is made possible by high-quality and selective microwave sensors. This technique utilizes the contrast between defected and normal tissues for detection. Various structures have been developed for detecting defects in human body tissues. The conductive nature of human tissue layers, particularly skin and muscle, results in higher attenuation of microwave power as it penetrates the tissue due to their higher water content. Therefore, precise detection requires high energy concentration and penetration. The literature has extensively studied the penetration depth and behavior of microwaves in the vicinity and inside the body tissues^1–3^. For instance,^1^ employed a split ring resonator (SRR) probe to investigate the penetration depth of microwaves in the thigh and distal femur bones while evaluating the recovery of extremity trauma patients. The study emphasized higher attenuation in tissues with high water content. Similarly,^2^ investigated the effect of different thicknesses of body layers such as fat and muscle on penetration depth using two multilayer SRR probes. Two adjacent SRR probes were used to investigate damages and changes in internal layers of human body tissues while studying the influence of fat and muscle layers on EM wave propagation. The study concluded that thicker fat layers retain more EM energy in body tissues. However, SRR probes suffer from problems such as highly sensitive performance to split gap capacitance and low accuracy in localizing defects in human body tissues. Small changes around the split gap can affect detection efficiency and power concentration in the structure, making this type of sensor unsuitable for raster scanning damaged body tissues.

Microstrip patch sensors have also found applications in the field of biomedical research. In^3^, a microstrip patch antenna array was evaluated as a screening sensor for osteoporosis, with a focus on miniaturization, and improvement of radiation characteristics and RF signal reception. Other studies^4,5^ have explored the use of raster scanning with microstrip patch antennas for bone fracture detection. These studies analyzed the reflection coefficients of the antennas to detect bone damage in a multilayer tissue model. Results showed that there was a small difference in reflection loss between normal bone and fractured bone. However, microstrip patch antennas can be limited by their low power concentration ability and require miniaturization for optimal performance.

Waveguide probes are another type of sensor used in microwave imaging, but their bulky structure and high fabrication costs limit their application in near-field probes. In^6^, a near-field rectangular waveguide probe was used to monitor bone fracture healing by modeling the fracture as a discontinuity between adjacent tissues.

Since biomedical sensors deal with human life, it is crucial to validate their performance through various experimental tests to ensure their quality, safety, and effectiveness. Mimicking phantoms are often used for accurate validation of the effect of EM waves on the human body. However, animal tissues such as bovine and pig are sometimes utilized^7^. It is important to note that when using animal tissues, neglecting tissue conductivity due to dehydration may lead to blind spots in the results obtained. Therefore, realized phantoms are considered the best choice for accurately evaluating sensor performance on the human body. To validate sensor performance, liquid, semi-solid, and solid phantoms with electrical properties similar to the human body are properly realized^8–10^.

When designing an optimal biomedical sensor, safety considerations including operating frequency, overall dimensions, impedance matching on body tissues, etc., should be considered. The difference in dielectric constants between air and skin, as the first layer of body tissue, causes significant power reflection, necessitating an interface matching medium between the sensor and tissues. Various anti-reflection coatings have been proposed in the literature, including ring and cross-shaped Frequency Selective Surfaces (FSS)^11,12^, dielectric-loaded waveguide structures^13^, and Double Negative (DN) dielectric lenses^14^. Reactive Impedance Surfaces (RIS) and Artificial Magnetic Conductors (AMC) are also commonly used techniques in microwave sensors and antennas to increase bandwidth^15^ or reduce size^16^. In^15^, the bandwidth of a patch antenna is increased using RIS. Additionally, the advantages of inductive RIS in sensor miniaturization are also evaluated. The application of AMCs in reducing size and improving radiation efficiency of biomedical sensors is discussed in^16^, where a uniplanar AMC-based patch antenna is used for breast tumor detection.

Combining miniaturization techniques with a dielectric lens can enhance the performance of patch resonators in biomedical imaging applications, improving resolution and accuracy. This study proposes a novel dual-polarized patch sensor equipped with a RIS layer and an inhomogeneous plano-concave dielectric lens for detecting fractures in human bones. The RIS layer is placed under the square patch to reduce path size and increase bandwidth, while the plano-concave dielectric lens, which is compatible with the curvature of human tissues, is positioned between the patch sensor and skin layer to improve EM energy penetration into the body. Two sensors, as a transmitter and a receiver, are placed opposite each other on the tissue and move simultaneously to detect bone fractures and monitor healing progress through simulated and measured scattering parameters and received power.

The scattering parameters are used to construct images from fractured area. The performance of the proposed transceiver system is validated through experimental measurements using a gelatin-based semi-solid phantom that simulates a human body. This system provides precise localization and orientation detection through the dual polarization capability, higher energy concentration, sharp images, the ability to detect narrow cracks (at least 1 mm), and a body-matched structure.

This paper is structured as follows: first, the design of the proposed sensor and phantom are presented. Next, the evaluation results of the sensor on a gelatin-based semi-solid phantom, which simulates a human body, are discussed through simulation and measurement. The mechanism of fracture detection is also explored. Finally, the conclusion section analyzes the ability of the sensor in fracture detection and the accuracy of images constructed from raster scan of human arm.

## Methods of design

### Design procedure of the transceiver sensor system

**Figure 1:**
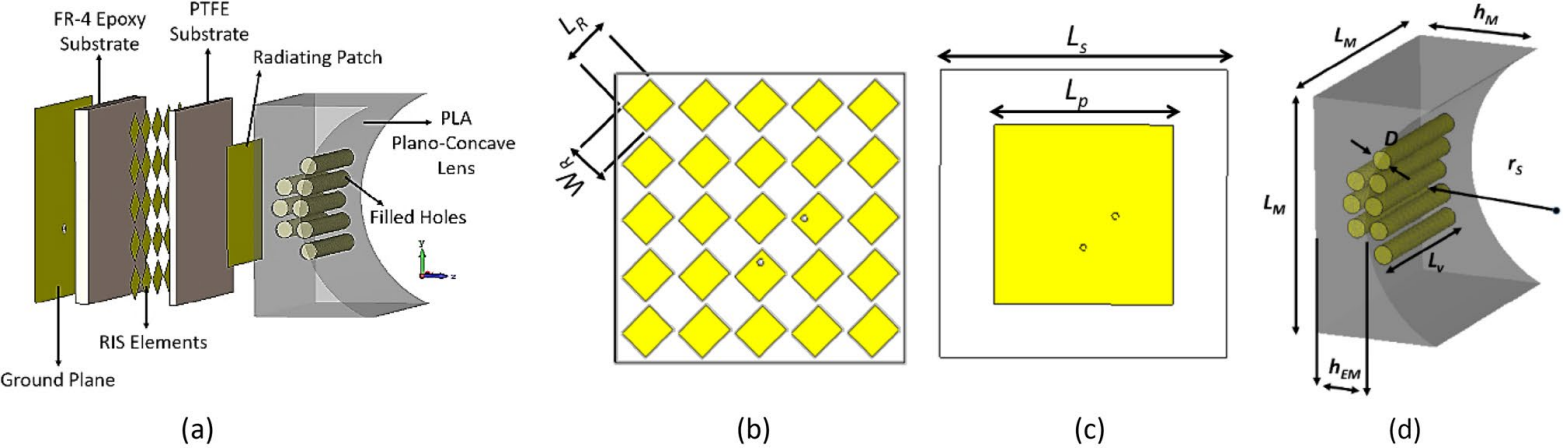
Geometry of the proposed sensor. (**a**) 3D model, (**b**) RIS elements, (**c**) radiating patch, (**d**) superstrate lens structure.

The proposed multilayer dual-polarized sensor comprises a square radiating patch designed on PTFE substrate with *ε*_*r*_ = 2.1, tan*δ* = 0.0002, and thickness of 1.5 mm, placed on a RIS layer, as shown in Fig. 1. The RIS layer is an array of rectangular patches printed on FR-4 substrate with *ε*_*r*_ = 4.4, tan*δ* = 0.02, and thickness of 3.2 mm. To enhance the energy transfer into body tissue and minimize reflection, an inhomogeneous plano-concave dielectric lens made of Polylactic Acid (PLA) is positioned on top of the radiating patch as a matching interface. The shape of the lens is designed to be compatible with the body’s curvature. Dual-polarized operation is achieved by exciting the sensor through two orthogonal coaxial cables. The parameters and orientation of the radiating patch and RIS elements are demonstrated in Fig. 1b,c. Figure 1d shows eight holes in the middle of the lens superstrate with a diameter of 4 mm filled with a material having electrical properties of *ε*_*r*_ = 5.28 and *σ* = 0.1 (s/m). These holes concentrate more energy in the center of the sensor to increase penetration depth and improve detection accuracy for bone cracks.

Patch sensors have limited use in biomedical applications due to their large dimensions, and low energy concentration and penetration in body tissues. However, in this study, various techniques are investigated to address these limitations. One such technique involves the use of a RIS layer, which reduces the overall size of the sensor and improves detection and localization accuracy. The rectangular elements of this layer are rotated 45° along their axes to ensure similar reflection phases for both vertical and horizontal polarizations. Table 1 summarizes the dimensions of the sensor, while subsequent sections will provide detailed discussions on the design of its various layers.

**Table 1:** Design parameters of the proposed sensor (unit: mm).

*L*_*R*_	*W*_*R*_	*L*_*s*_	*L*_*p*_	*D*	*L*_*M*_	*h*_*M*_	*h*_*EM*_	*r*_*S*_	*L*_*v*_
6.4	5.6	46	28	4	50	28	11.4	27	30

#### Design of the reactive impedance surface (RIS) layer

**Figure 2:**
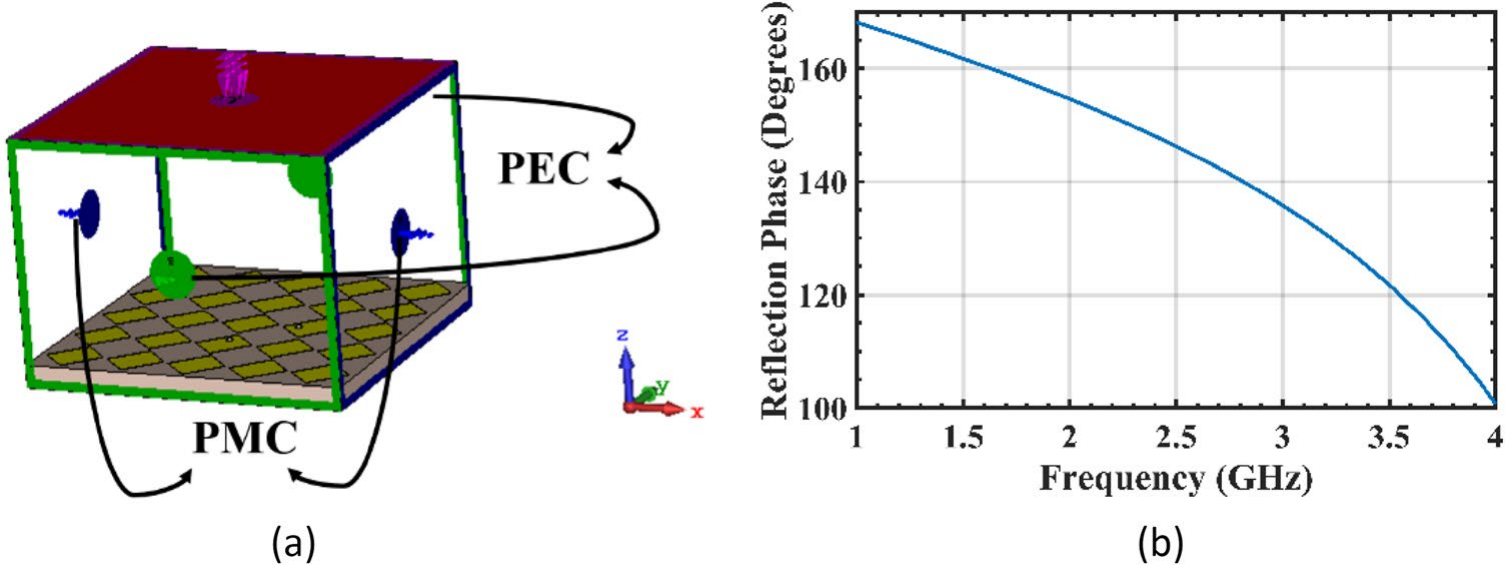
(**a**) Simulation setup for measuring the reflection phase of the RIS structure, (**b**) reflection phase diagram.

To achieve precise detection of narrow cracks with a width of approximately 1 mm, the sensor utilizes an RIS layer located at the back of the radiating patch to reduce its overall dimensions. The pure inductive property of the RIS cancels out the capacitive near-field radiation of the patch, resulting in an increase in radiation efficiency. The reflection phase of an inductive RIS structure must be between 180° (as a Perfect Electric Conductor—PEC) and 0° (as an Artificial Magnetic Conductor—AMC). The reflection phase from a RIS illuminated by a normal incident wave can be calculated using Eq. (1)^15^:

1 $$\phi = {\text{Im}} \left[ {Ln\frac{{Z_{s} - \eta }}{{Z_{s} + \eta }}} \right]$$

where *Z*_*s*_ is the impedance of the RIS layer and *η* is the intrinsic impedance of free space. The reflection phase calculated from Eq. (1) yields an inductive reactance from the RIS. This RIS structure located between the radiating patch and its ground plane produces additional inductive reactance, reducing the resonant frequency of the sensor and leading to a reduction in size. The array of 5 × 5 rectangular RIS elements are illustrated in Fig. 1b. The simulation setup to obtain the reflection phase of the proposed RIS layer is shown in Fig. 2a, and the reflection phase diagram of the structure is shown in Fig. 2b. The proposed RIS layer exhibits a reflection phase of approximately 145° at the operating frequency of *f*_o_ = 2.45 GHz, which falls between 0° (AMC) and 180° (PEC), confirming Eq. (1).

#### Design of the plano-concave inhomogeneous dielectric lens

According to Snell’s law, when electromagnetic waves pass from a less dense medium to a denser one, they bend towards the normal to the boundary. The high contrast between the permittivity of the patch sensor and the body tissue, particularly the skin as the first layer of the body with *ε*_*r*_ = 38, results in a significant reflection at the boundary. To mitigate this reflection and enhance the power transfer, an interface dielectric material is used as a matching medium. Here, a plano-concave lens made of Polylactic Acid (PLA) with *ε*_*r*_ = 3 and *σ* = 0.0001 (s/m) is employed as the superstrate and matching medium between the patch and the body. The gradual increment in permittivity helps in reducing undesired reflections. The EM wave initially enters into the PLA material from the radiating patch and then proceeds to penetrate into the body.

To achieve higher energy concentration and deeper penetration of the EM waves, it is important to minimize scattering and direct the waves to propagate normally at the interface between the lens and the human body. This can be accomplished by integrating a few dielectric-filled holes into the center of the lens. These holes converge the waves, resulting in enhanced precision in detecting cracks and fractures. The filling material used in these holes is similar in properties to the fat tissue in the human body, with greater electrical permittivity and conductivity than the surrounding PLA medium. This contributes to greater wave concentration in the center of the lens.

**Figure 3:**
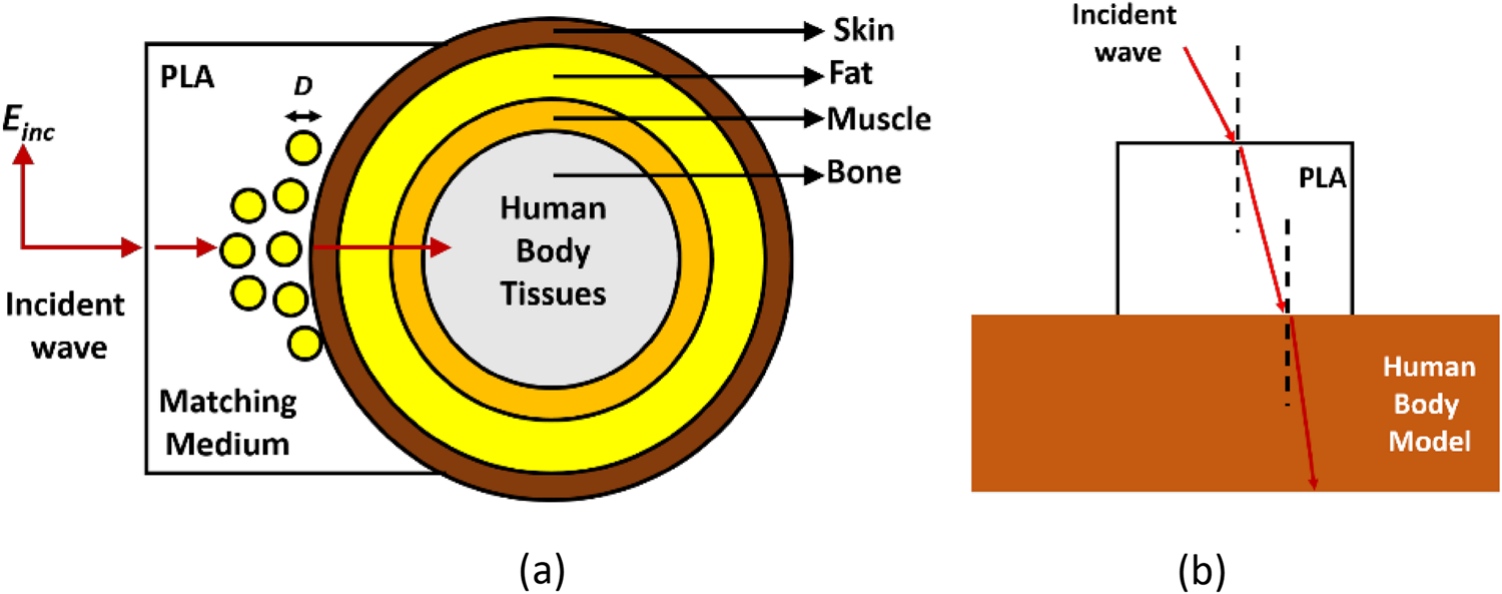
Transmission of the incident wave between different layers. (**a**) Side view, (**b**) top view.

The human body can be represented as a multilayer dielectric medium, comprising skin, fat, muscle, and bone layers. Skin and muscle tend to dissipate EM power more than fat and bone due to their higher water content. In the simulation, a cylindrical multilayer structure is used to model the truncated arm. Figure 3a,b provides a visual representation of how the EM waves radiated from the transmitter penetrates the internal layers of the body, making it possible to detect bone fractures.

To achieve a gradual increase in permittivity, a plano-concave shape is selected for the matching lens. The ideal permittivity for the matching medium is calculated as the geometric mean of the permittivity of the two mediums on either side of the lens, with a thickness equivalent to λ/4. In this study, the permittivity of PLA is brought closer to the calculated ideal value by the dielectric vias embedded in the lens, as shown in Fig. 1d, assuming an incident wave at the resonant frequency of the proposed sensor (2.45 GHz). The thickness of the lens is slightly greater than λ/4 to account for the difference between the permittivity of the real PLA and the ideal matching material. The number of filled holes can be optimized by evaluating insertion loss and EM energy concentration.

In this study, eight vias are embedded in the lens, arranged in two consecutive rows. As the size of these vias is less than the wavelength (*πD*/*λ* < 1), they can be considered as electric dipoles that generate electric momentums. As a result, the equivalent dielectric constant of the proposed structure can be calculated using the Discrete Dipole Approximation (DDA) method, as follows^17^:

2 $$\varepsilon_{eff} = \varepsilon_{H} + \frac{{3F. \, \varepsilon_{H} . \, \left( {\frac{{\varepsilon_{r} - \varepsilon_{H} }}{{\varepsilon_{r} + 2\varepsilon_{H} }}} \right)}}{{1 - F. \, \left( {\frac{{\varepsilon_{r} - \varepsilon_{H} }}{{\varepsilon_{r} + 2\varepsilon_{H} }}} \right)}}$$

where *ε*_*r*_ is the dielectric constant of the filled vias, *ε*_*H*_ is the dielectric constant of PLA as the surrounding medium, and *F* is the ratio of the volume of vias to the portion of PLA volume involved in the propagation of EM waves. The estimated volume ratio for this design is approximately *F ≈* 0.14, resulting in an effective dielectric constant of* ε*_*eff*_ = 3.3. Therefore, the use of vias in the matching lens leads to an increase in its effective permittivity. If we consider only the volume of the lens located directly in front of the radiating patch as the effective radiation volume, the effective dielectric constant still rises to *ε*_*eff*_ = 3.94 with a volume ratio of *F ≈* 0.32.

**Figure 4:**
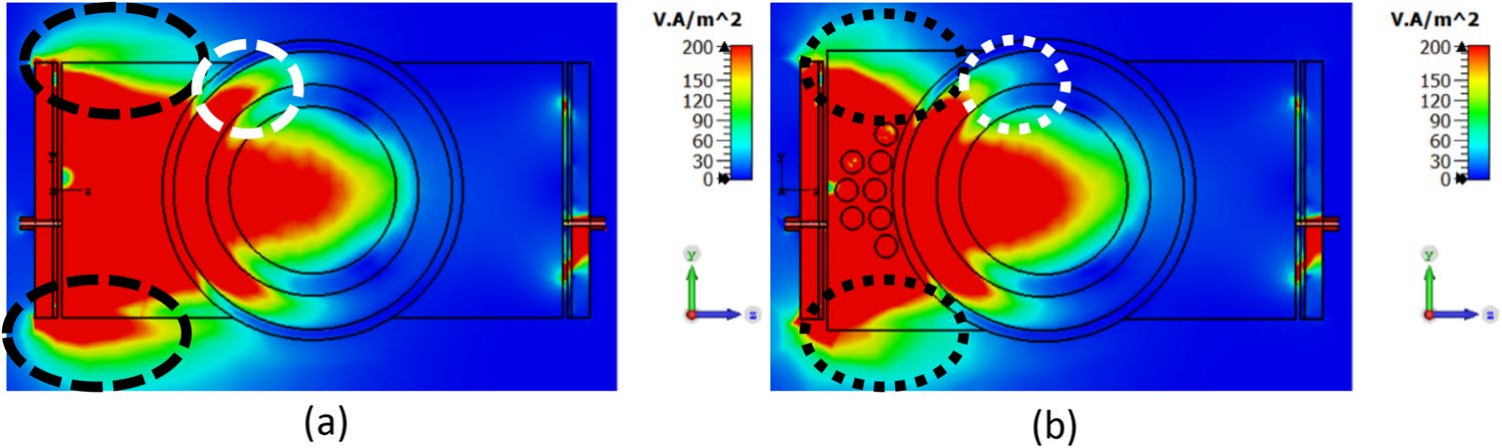
The EM power flow from the transmitter to the receiver sensor through the body tissue with (**a**) homogenous lens without vias, (**b**) inhomogeneous modified lens.

The more conductive nature of these vias compared to PLA directs the EM waves from the edges to the center of the lens. Figure 4 shows a comparison between the penetration of EM fields with and without dielectric vias, where the modified lens with vias concentrates more power in the center of the structure and around the crack than the homogenous lens (without vias). The marked regions in Fig. 4 demonstrate the improved power concentration around the crack achieved with the vias, reducing power spread around in the fat layer. As the EM waves approach the skin layer, the number of vias increases, allowing the power penetrating the body tissue to gradually sense the inhomogeneity of the lens, resulting in reduced reflection loss.

The proposed matching structure offers lower attenuation compared to liquid matching mediums like deionized water. Solid structures are also generally easier to fabricate and use in practical applications, making them a preferred choice.

#### EM wave propagation analysis in human body tissues

One of the major obstacles in developing sensors for detecting bone fractures is the conductive and lossy properties of human tissues that have high water content. This is particularly challenging for bone, which is located deep within a multilayer tissue structure and experiences significant attenuation of incident power due to the deflection of EM waves caused by the dielectric contrast between tissue layers. Previous researches have utilized approximate mathematical formulas to evaluate the attenuation of power in human tissues, providing insights into power attenuation across different tissue layers. The mathematical calculations presented in^18,19^ can be valuable references for wireless deep implant communication applications, including the design of antennas, frequency, power, direction, and wireless power provision.

The complex propagation constant in each layer is given by^18^:

3 $$\begin{gathered} \gamma = \alpha + j\beta \hfill \\ \alpha = \omega \sqrt {\frac{\mu \varepsilon }{2}\left[ {\sqrt {1 + (\frac{\sigma }{\omega \varepsilon })^{2} } - 1} \right]} \, \hfill \\ \beta = \omega \sqrt {\frac{\mu \varepsilon }{2}\left[ {\sqrt {1 + (\frac{\sigma }{\omega \varepsilon })^{2} } + 1} \right]} \, \hfill \\ \varepsilon = \varepsilon_{0} (\varepsilon^{\prime} - j\varepsilon^{\prime\prime}) = \varepsilon_{0} (\varepsilon^{\prime} - j\frac{\sigma }{{\omega \varepsilon_{0} }}) \hfill \\ \end{gathered}$$

where $\alpha$ and $\beta$ are attenuation coefficient and phase constant, respectively, $\omega$ is angular frequency, and $\sigma$, $\varepsilon$, and $\mu$ are the conductivity, permittivity and permeability of the layers, respectively. To determine the amplitude difference in the transmitted signals (|S_21_|) between healthy and fractured bones, Eq. (4) is used^20^:

4 $${{Attenuation}} = {{exp(}} - \upalpha {{t) [dB]}}$$

where *t* is the thickness of each layer of body tissue.

For biomedical applications, such as body area networks (BAN) and bone fracture detection systems, these equations are useful for analyzing attenuation and losses in different layers of the human body^18^. The accuracy of this approximation is supported by simulations conducted on tissues with known loss properties, as well as experimental measurements performed on a fabricated arm phantom in this study. Table 2 displays the electrical properties^7,21^ and estimated attenuation of EM power in each layer of human tissue. Taking into account that the receiver is located on the opposite side of the transmitter sensor and the power passes through each layer twice, the loss calculations double the thickness of each layer. Tissues with high water content tend to attenuate more power.

**Table 2:** Electrical parameters and loss factors of human body tissues.

Body tissue	$\upepsilon^{\prime}$	$\upepsilon^{\prime\prime}$	$\upalpha$	Conductivity (S/m)	Thickness (mm)	Attenuation (dB)	Approximate attenuation (%)
Skin	38	11	43.7	1.46	2	− 0.77	20
Fat	5.28	0.8	8.52	0.11	6	− 0.48	10
Muscle	52.7	13	43.9	1.74	4	− 1.5	30
Bone	11.4	3	21.9	0.39	15	− 3	50
Blood	58.2	18.7	63.5	2.54	15	− 8.3	85

According to Table 2, the transmission of power through the human body results in significant losses. The 4 mm skin layer attenuates 20% of the input power, leaving 80% remaining. Of this remaining power, 10% is attenuated by the 12 mm fat layer resulting in an overall loss of 28%. The subsequent muscle and bone layers continue to exhibit similar losses. Based on these calculations, it is determined that when EM waves propagate through a healthy bone, approximately 75% of its power is lost. When the bone is damaged, and a layer of blood fills the cracked region, the loss of power increases to approximately 92.5%. Consequently, the amount of received power on the opposite side of a healthy bone would be 17.5% greater than that of the fractured bone.

It is important to note that our calculations assumed the entire power is transmitted through the blood within a crack. However, in reality, particularly in narrow cracks, most of the power is transmitted through the bone tissue with only a small amount passing through the blood-filled crack. Therefore, the actual difference in received power between a healthy bone and a fractured bone may be lower than the calculated value of 17.5%. For instance, in a bone with a 1 mm-wide crack, the simulated loss (|S_21_|) indicates a difference of approximately 4.4% when compared to a normal (healthy) bone. The wider the crack, the more pronounced the contrast between the blood-filled crack and the bone tissue, resulting in a big difference in the received power compared to a normal bone.

In addition, our study also includes a near-field analysis. In near-field analysis, the scattering occurs at the interfaces between different tissue layers must be taken into account. This involves the interaction of standing waves at the interfaces between layers, as well as the transmitted waves. This concept is related to the theory of surface waveguides, where EM waves travel along interfaces between different media. Here, to evaluate the effects of EM radiation on cylindrical media, like the human arm, the discussion focuses on the homogenous, isotropic, and source-free wave equations. For simplicity of calculations, the length of the cylinder (representing the arm) is assumed infinite.

**Figure 5:**
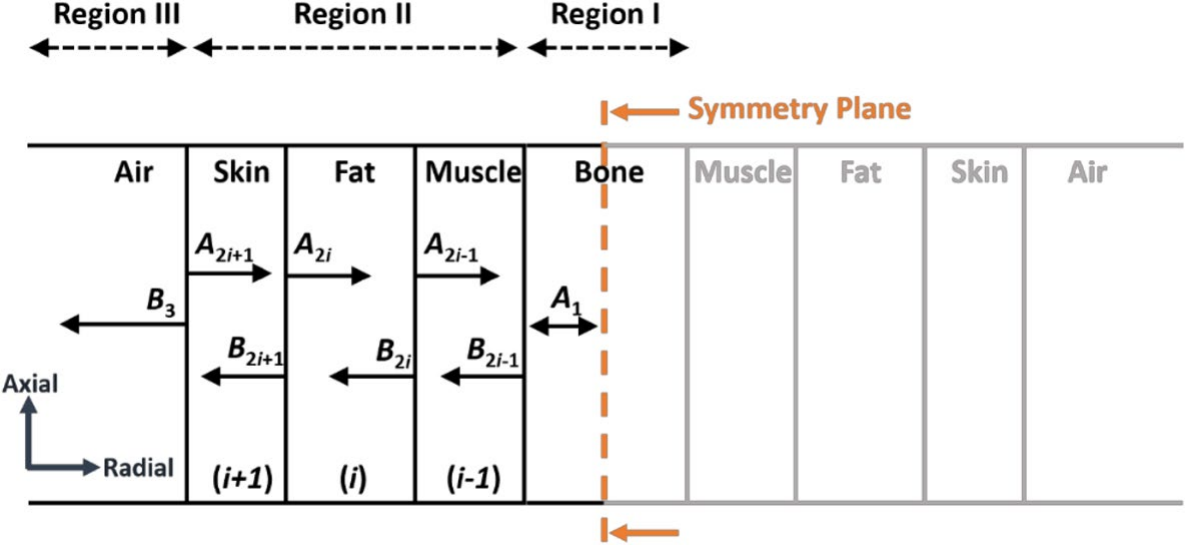
Different types of regions classified to evaluate the scattering of EM waves in human body tissues.

We utilized a homogeneous multilayer tissue model, with the human arm as the subject of research. The model excluded certain internal tissues such as blood vessels and nerves, and simplified the arm into four tissues: skin, fat, muscle, and bone. To simplify the analysis, we focused on normal incidence and considered the interfaces between tissues as equivalent planes, as shown in Fig. 5. The problem environment consists of five layers: air, skin, fat, muscle, and bone. We defined the center of the bone tissue as the origin of the coordinate system. Based on the characteristics of EM wave propagation in multilayer media, we classified these layers into three different regions based on the type of propagated waves^22^.

In region I, for the bone as the innermost layer, it is assumed that the wave is completely reflected at its center, leading to the formation of a standing wave within the bone. The axial direction of the EM fields can be expressed in terms of Bessel function due to the singularity of other types at the center of the bone (*r* = 0) as shown in Eq. (5)^22^:

5 $$\left(\begin{array}{c}{\text{E}}_{\text{ax}}\\ {\text{H}}_{\text{ax}}\end{array}\right)\text{=}{\bar{\text{A}}}_{1}{{\text{J}}}_{\text{n}}\left({\text{k}}_{{1}{\text{r}}}{\text{r}}\right)$$

where ${\bar{\text{A}}}_{1}$ and *k*_1*r*_ are, respectively, the amplitude of standing wave and radial propagation constant in region I. On the other hand, the arm tissues, including skin, fat, and muscle, are considered as region II, where both standing and transmitted waves exist in these three layers. The electric and magnetic fields can be mathematically expressed by a combination of Bessel and Hankel functions as illustrated in Eq. (6):

6 $$\left(\begin{array}{c}{\text{E}}_{\text{ax}}\\ {\text{H}}_{\text{ax}}\end{array}\right)\text{=}{{\bar{\text{A}}}_{{2}{\text{i}}}{\text{J}}}_{\text{n}}\left({\text{k}}_{{2}{\text{ir}}}{\text{r}}\right)\text{+}{\bar{\text{B}}}_{{2}{\text{i}}}{\text{H}}_{\text{n}}^{(1)}\left({\text{k}}_{{2}{\text{ir}}}{\text{r}}\right)$$

where ${\bar{\text{A}}}_{{2}{\text{i}}}$ and ${\bar{\text{B}}}_{{2}{\text{i}}}$ denote the amplitudes of standing and transmitted waves, respectively, and *k*_2*ir*_ is radial propagation constant in region II. Here, *i* represents the fat layer, the (*i* − 1) muscle layer, and the (*i* + 1) skin layer in region II.

In region III, which represents the air layer, only a transmitted EM wave is present and it can be expressed using Hankel function as shown in Eq. (7).

7 $$\left(\begin{array}{c}{\text{E}}_{\text{ax}}\\ {\text{H}}_{\text{ax}}\end{array}\right)\text{=}{\bar{\text{B}}}_{3}{{\text{H}}}_{\text{n}}^{(1)}\left({\text{k}}_{{3}{\text{r}}}{\text{r}}\right)$$

By applying the principle of continuity for the tangential component of electric field and the normal component of magnetic flux density at the interface between two adjacent media, we can derive the transmission matrix ${\bar{\text{T}}}_{\text{ij}}$ (or ${\bar{\text{T}}}_{\text{ji}}$) and reflection matrix ${\bar{\text{R}}}_{\text{ij}}$ expressions for the interface between layers *i* and *j*, as presented in Eqs. (8) and (9), respectively^23^:

8 $$\begin{gathered} \overline{T}_{ij} = \frac{2\omega }{{\pi k_{ir}^{2} a}}\overline{D}^{ - 1} \left[ {\begin{array}{*{20}c} {\varepsilon_{i} } & 0 \\ 0 & { - \mu_{i} } \\ \end{array} } \right] \hfill \\ \overline{T}_{ji} = \frac{2\omega }{{\pi k_{jr}^{2} a}}\overline{D}^{ - 1} \left[ {\begin{array}{*{20}c} {\varepsilon_{j} } & 0 \\ 0 & { - \mu_{j} } \\ \end{array} } \right] \hfill \\ \end{gathered}$$

9 $$\begin{gathered} \overline{R}_{ij} = \overline{D}^{ - 1} \left[ {H_{n}^{(1)} \left( {k_{ir} a} \right)\overline{H}_{n}^{(1)} \left( {k_{jr} a} \right) - H_{n}^{(1)} \left( {k_{jr} a} \right)\overline{H}_{n}^{(1)} \left( {k_{ir} a} \right)} \right] \hfill \\ \overline{R}_{ji} = \overline{D}^{ - 1} \left[ {J_{n} \left( {k_{ir} a} \right)\overline{J}_{n} \left( {k_{jr} a} \right) - J_{n} \left( {k_{jr} a} \right)\overline{J}_{n} \left( {k_{ir} a} \right)} \right] \hfill \\ \end{gathered}$$

where *a* is the radius of each layer in the arm tissue. ${\bar{\text{T}}}_{\text{ij}}$ represents the EM wave propagating in layer “*i*” with the propagation constant of *k*_*ir*_, which is transmitted to an arbitrary layer of “*j*”. Similarly, ${\bar{\text{T}}}_{\text{ji}}$ evaluates the EM wave propagating in layer “*j*” with the propagation constant of *k*_*jr*_, which is transmitted to an arbitrary layer of “*i*”. This notation is also applicable to the reflection matrix in Eq. (9). The functions of D, ${\stackrel{\mathrm{-}}{\text{J}}}_{\text{n}}\left({\text{k}}_{\text{ir}}{\text{r}}\right)$ and ${\stackrel{\mathrm{-}}{\text{H}}}_{\text{n}}^{(1)}\left({\text{k}}_{\text{ir}}{\text{r}}\right)$ can be calculated using Eq. (10).

10 $$\begin{gathered} \overline{D} = \left[ {\overline{J}_{n} \left( {k_{ir} a} \right)H_{n}^{(1)} \left( {k_{jr} a} \right) - J_{n} \left( {k_{ir} a} \right)\overline{H}_{n}^{(1)} \left( {k_{jr} a} \right)} \right] \hfill \\ \overline{J}_{n} \left( {k_{ir} r} \right) = \frac{1}{{k_{ir}^{2} }}\left[ {\begin{array}{*{20}c} {j\omega \varepsilon_{i} k_{ir} rJ_{n}^{^{\prime}} \left( {k_{ir} r} \right)} & { - nk_{z} J_{n} \left( {k_{ir} r} \right)} \\ { - nk_{z} J_{n} \left( {k_{ir} r} \right)} & { - j\omega \mu_{i} k_{ir} rJ_{n}^{^{\prime}} \left( {k_{ir} r} \right)} \\ \end{array} } \right] \hfill \\ \overline{H}_{n}^{(1)} \left( {k_{ir} r} \right) = \frac{1}{{k_{ir}^{2} }}\left[ {\begin{array}{*{20}c} {j\omega \varepsilon_{i} k_{ir} rH_{n}^{^{\prime}(1)} \left( {k_{ir} r} \right)} & { - nk_{z} H_{n}^{(1)} \left( {k_{ir} r} \right)} \\ { - nk_{z} H_{n}^{(1)} \left( {k_{ir} r} \right)} & { - j\omega \mu_{i} k_{ir} rH_{n}^{^{\prime}(1)} \left( {k_{ir} r} \right)} \\ \end{array} } \right] \hfill \\ \end{gathered}$$

After obtaining the transmission and reflection matrices, it becomes feasible to establish the relationship between the amplitudes of standing waves and transmitted waves. The numerical methods presented in^21^ were utilized to compute these relationships, which were then employed to examine the near-field propagation in a model of the human arm.

### Realized human arm phantom

**Figure 6:**
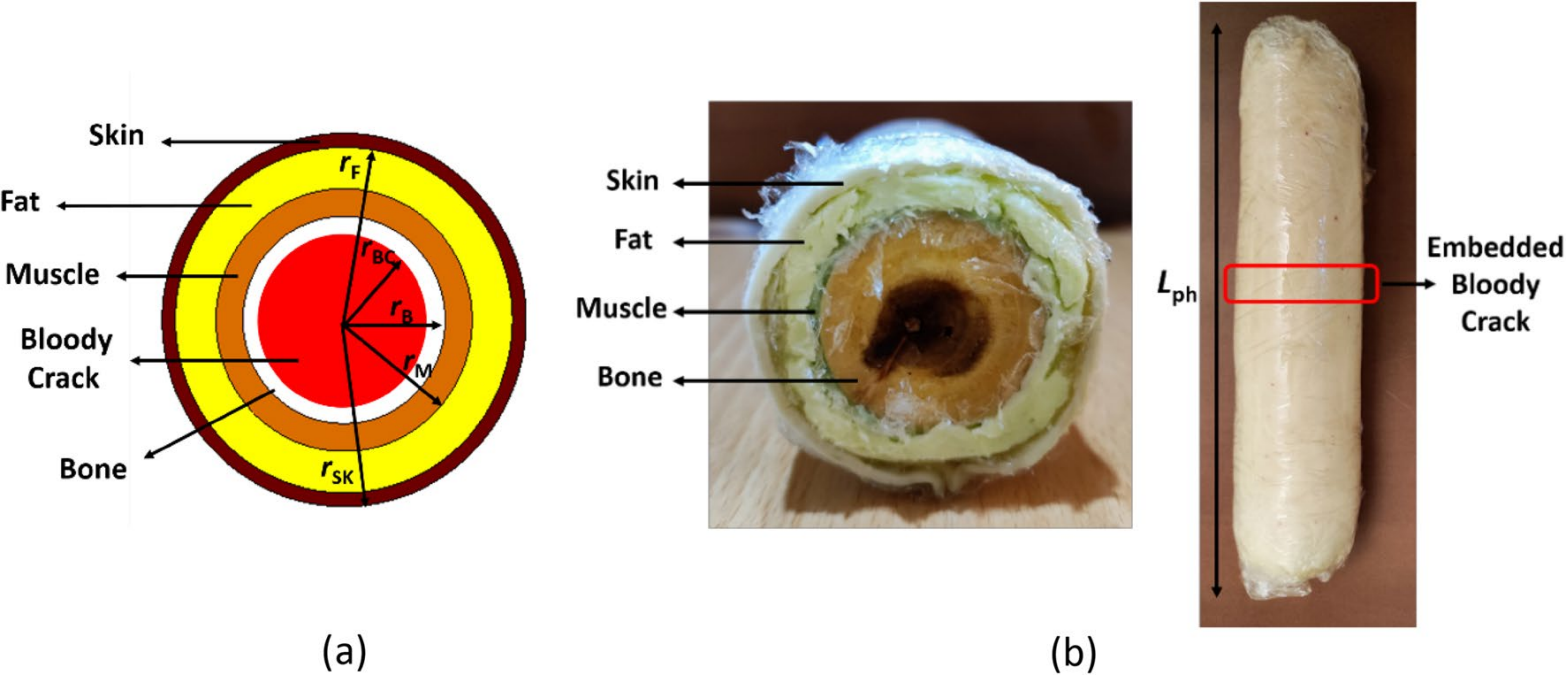
Multilayer human body model. (**a**) Truncated simulation model, (**b**) fabricated realized phantom.

Non-destructive performance of the proposed sensor must be validated by experimental measurements. Here, to emulate a real human arm with a fractured bone, a gelatin-based semi-solid structure mimicking a phantom is introduced, where its reliability and similarity to a real body tissue are experimentally tested. The simulated multilayer model of human arm and the fabricated phantom are shown in Fig. 6a,b, respectively, where both of them include a blood-filled fractured area. The electrical properties of the layers used in both the simulation and fabrication of the phantom corresponded to those of a living human. The dimensions of the fabricated phantom are outlined in Table 3.

**Table 3:** Dimensions of the realized arm phantom (unit: mm).

*r*_BC_	*r*_B_	*r*_M_	*r*_F_	*r*_Sk_	*L*_ph_
15	15	19	25	27	280

The improved gelatin-based cylindrical phantom is made with a mixture of deionized water, pure vegetable oil, soap, gelatin leaves and salt. To fabricate each layer of the phantom, such as skin, fat, muscle, and blood, the necessary compounds and their amounts were precisely derived from^10^, which are summarized in Table 4. To ensure the structural integrity and stability of the cylindrical phantom, a thin layer of cellophane is applied to tighten it. This layer not only helps maintain the shape of the phantom but also acts as a protective barrier, reducing the interaction of the ingredients with the air and preserving their freshness. In addition, a water-saturated wood was also used to represent the bone tissue due to its similarity to human bone^24^. The proposed detection mechanism in this study involves comparing transmission coefficients of normal and fractured bones (amplitude and phase) and constructing bone images by analyzing their difference. This approach is not expected to be significantly affected by minor variations in permittivity and conductivity of each tissue layer.

**Table 4:** Ingredients of the realized body phantom.

Tissue	Ingredient				
	Gelatin (gr)	Deionized water (gr)	Oil (gr)	Salt (gr)	Soap (gr)
Skin	17	100	35	0.8	25
Fat	14	60	210	0	10
Muscle	17	100	20	1	20
Blood	8	50	10	0.6	10

**Figure 7:**
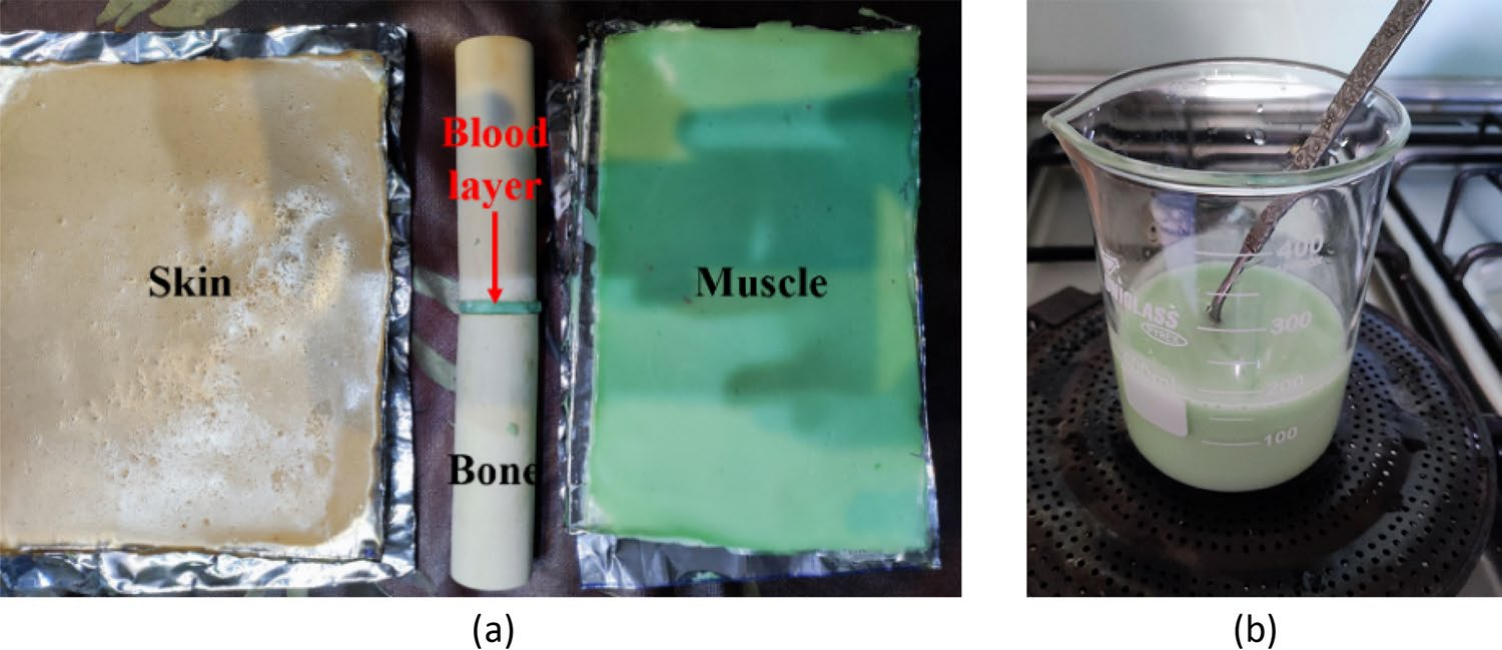
Phantom fabrication. (**a**) Filling the broken region with blood, (**b**) homogenization process.

In order to model a fractured region in bone tissue, an experimental setup was created using two pieces of water-saturated wood with a thin layer of blood embedded between them, as shown in Fig. 7a. The purpose of this blood layer is to replicate the presence of blood flowing out from damaged vessels in a real-life broken bone scenario. The mixture was homogenized by stirring it in a beaker, as demonstrated in Fig. 7b.

In the fabrication process, gelatin leaves were soaked in deionized water and then mixed with 50 g of deionized water in a boiler. The mixture was heated to 85 °C and stirred until it became gummy. After cooling to around 40 °C, the remaining water and salt were added while stirring slowly to avoid creating bubbles. Next, Fairy Platinum dishwashing liquid was added based on its ability to act as a solvent for water and canola oil^10^. Finally, pure vegetable oil was added and the mixture was stirred until it solidified over a few hours.

The gelatin allows the mixture to solidify, while salt is used to achieve the desired conductivity and oil content regulates the dielectric constant of each tissue layer. A higher oil content results in a lower permittivity, which is used to produce the fat layer. The cylindrical structure of the phantom was designed to provide a more realistic representation of the human arm than previous studies^8–10^, enhancing the accuracy and reliability of experimental measurements for evaluating fractures in the human arm.

## Results and discussion

### Performance evaluation of the proposed sensor on the human body

The path of EM waves propagation from the transmitter to the receiver of the sensor is demonstrated in Fig. 4. At the interface of skin and fat tissues, deviation of EM waves causes a decrease in accuracy of bone fracture detection due to transmission loss. For example, when a wave with an incident angle of θ_*i*_ = 10° propagates from the skin to the fat layer, it is transmitted with an angle of θ_*t*_ = 26.8°. This deviation from the normal direction is one of the main challenges in transmitting EM power in biomedical microwave sensors.

**Figure 8:**
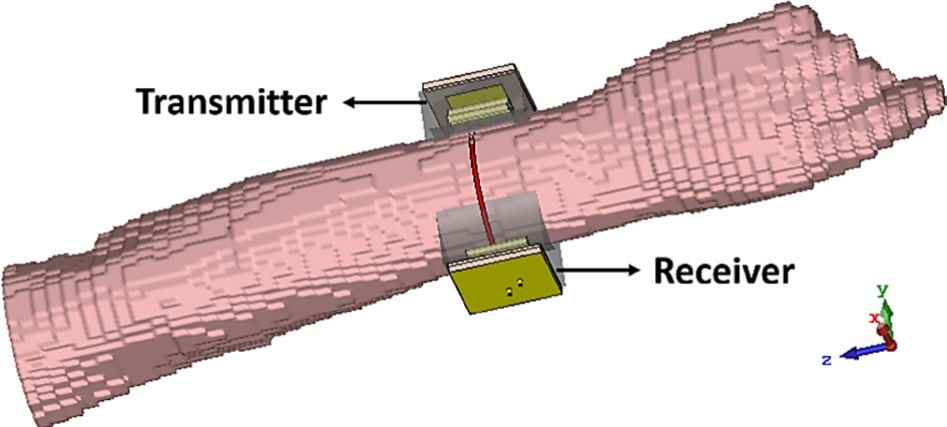
Geometry of the proposed system on human body.

The transceiver sensor is positioned on the body tissue model, and its performance is simulated using full-wave CST Microwave Studio and optimized to achieve desired reflection and insertion losses. The non-destructive nature of the proposed sensor in detecting and characterizing small changes in the human body makes it suitable for safe biomedical applications. Here, analysis and imaging are conducted based on measured parameters in the frequency domain to verify the possibility of bone fracture detection using highly sensitive microwave sensors. The orientation of sensors and system geometry are depicted in Fig. 8.

**Figure 9:**
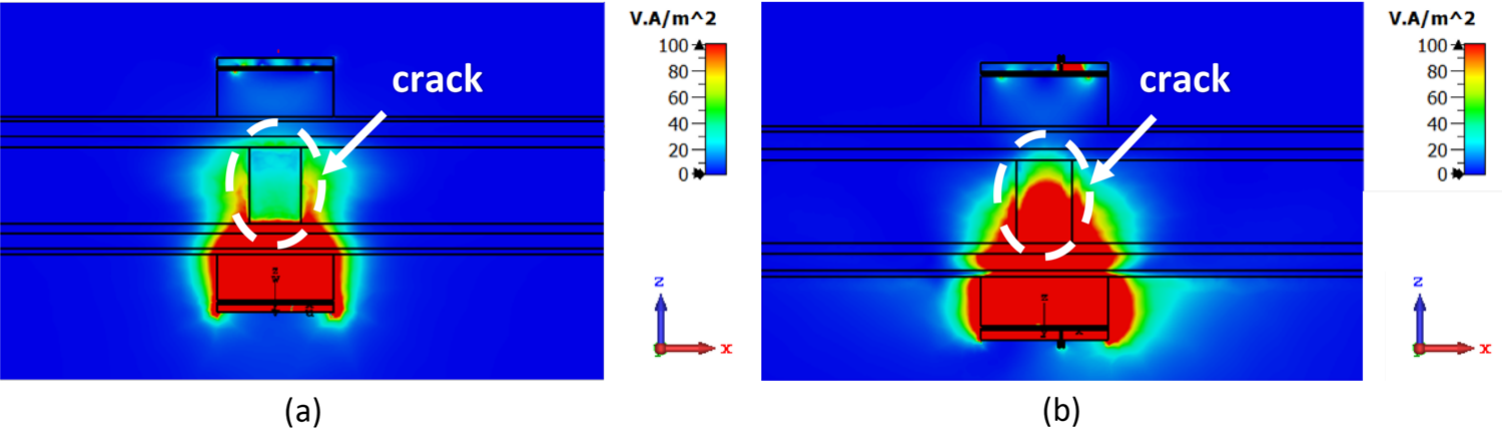
Power flow of EM waves in the human body when the incident E-field is (**a**) perpendicular to the crack orientation, (**b**) tangent to the crack orientation.

The polarization of the transmitted wave is another crucial parameter in fracture detection. To ensure accurate detection, the E-Field must be tangential to the boundary of the crack in the bone tissue. In NDT applications, when a crack occurs on a metallic surface, EM waves propagate through the step-like crack region, and the resonant frequency of the structure shifts down due to the increased path length of the current^25^. For dielectric mediums, as a dual counterparts of metallic structures, a tangential electric field on the crack surface is desired to increase interaction with the crack. Figure 9 illustrates the transmission of EM power from the transmitter to the receiver for two orthogonal polarizations. When the E-field is perpendicular to the crack, EM waves change their path and do not propagate through the cracked region, as shown in Fig. 9a. This configuration is less favorable for crack recognition. However, as shown in Fig. 9b, when the E-field is tangential to the crack orientation, most of the EM waves propagate through the cracked region, resulting in improved crack recognition and enhanced sensitivity of the sensor to fractures in bone tissue.

The proposed sensor system utilizes two orthogonal polarizations to enhance the precision of detecting and locating both transverse and longitudinal narrow cracks. The higher conductivity of blood in comparison to bone allows the blood layer within the crack to be considered as a dielectric waveguide. The contrast between the blood and bone tissues forms the basis for image construction through the measured scattering parameters, aiding in crack detection.

**Figure 10:**
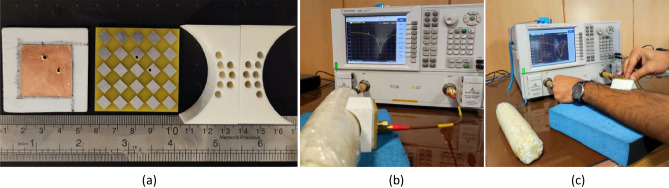
Experimental setup for measurement. (**a**) Different layers of the fabricated sensors, (**b**) measurements on the realized phantom, (**c**) measurements on the human arm.

**Figure 11:**
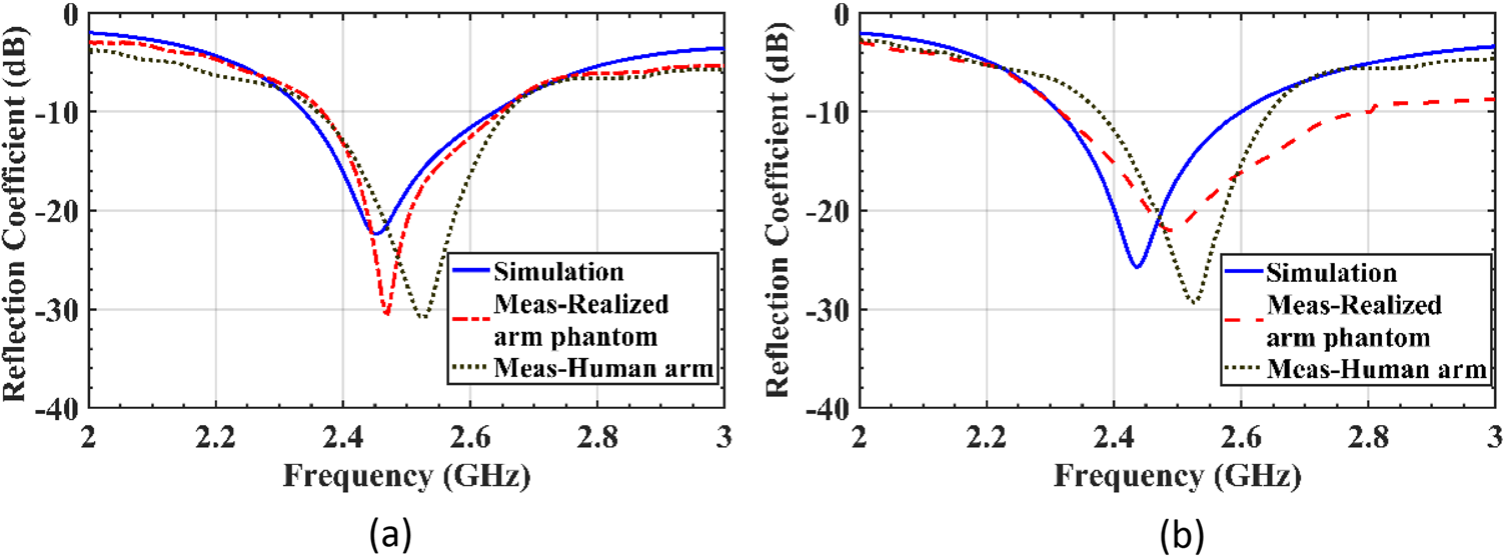
Comparison of the reflection coefficients of the sensor (|S_11_| or |S_22_|) when placed on a real human arm, a realized arm phantom, and a simulated model in the healthy region. The incident E-field is tested in two polarizations: (**a**) tangent to the crack orientation, (**b**) perpendicular to the crack orientation.

The transceiver sensors are fabricated and their performances are evaluated on both the realized phantom and a real human arm, as shown in Fig. 10a–c. The transmission and reflection coefficients are measured for both cases using an Agilent E8363C Network Analyzer. The simulated and measured reflection coefficients for both tangent and perpendicular propagation are presented in Fig. 11, indicating that the sensor system operates at a center frequency of 2.45 GHz with a bandwidth of 12.5%. The measured results demonstrate that the implemented arm phantom performs similarly to a real human arm, validating the effectiveness of the sensor system in a realistic setting. Furthermore, the comparison of the results indicate that the transceiver sensor is slightly sensitive to the presence of blood vessels, but this does not affect the constructed images of fractured bones since the presence of blood is included in the affected region.

### Detection mechanism and image construction

**Figure 12:**
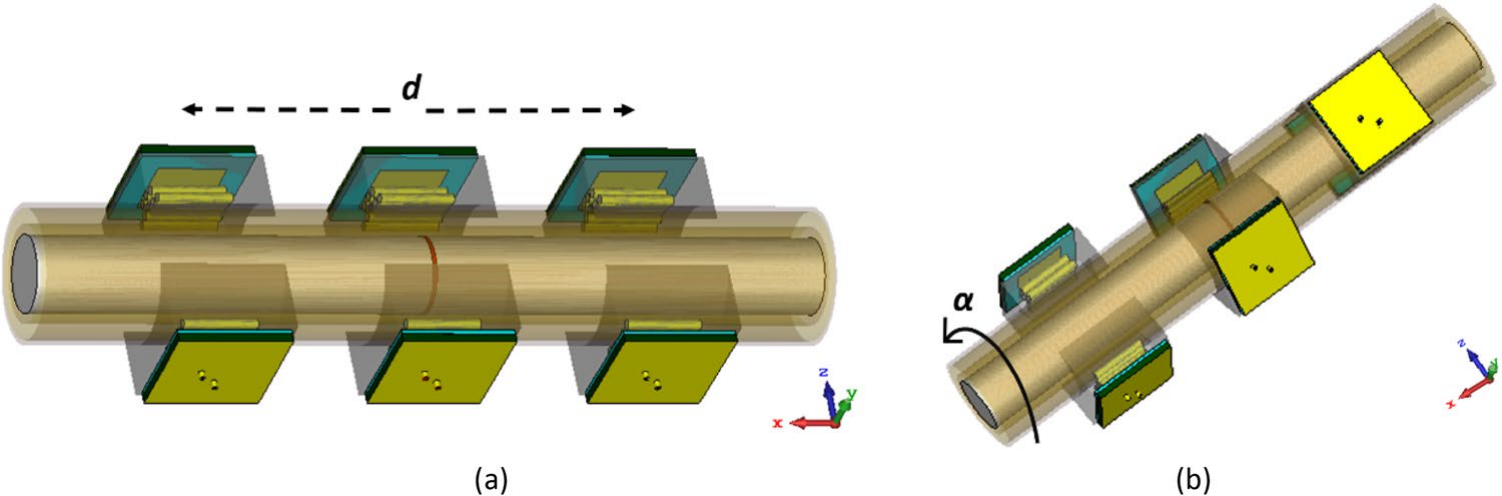
Raster scanning configuration. (**a**) Longitudinal scan, (**b**) rotational scan.

According to Fig. 12, two sensors of the transceiver system move together in a line to scan the entire arm, measuring the transmission loss between them to construct an image of the damaged bone tissue. The transceiver sensors perform simultaneous longitudinal and rotational scanning of the tissue, obtaining a dataset by raster scanning and measuring the scattering parameters. The amplitude and phase of the transmission coefficients provide valuable information about the location and width of the fractures, while reflection coefficients can also be used for image construction. The position of the transceiver sensors and scanning procedure for the body are illustrated in Fig. 12a,b. The measurements are carried out for both incident E-fields tangential and perpendicular to the crack orientation.

**Figure 13:**
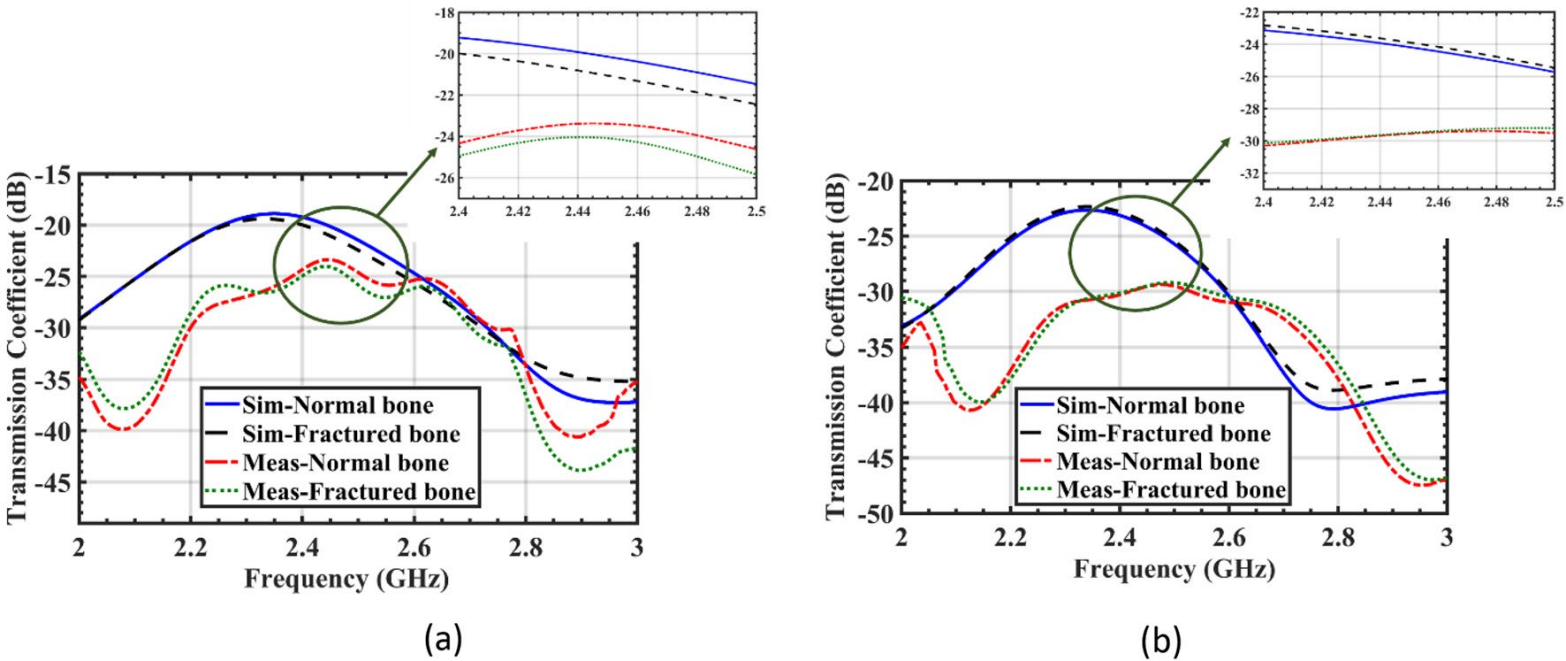
Comparison of the simulated and measured transmission coefficients (|S_21_|) of the sensor when placed on both the realized arm phantom and the simulated model with a 1 mm crack. The incident E-field is tested in two polarizations: (**a**) tangent to the crack orientation, (**b**) perpendicular to the crack orientation.

The evaluation of the sensor performance and the image construction are based on the difference between the amplitudes and the phases of the transmission coefficients (S_21_) of normal and cracked bones. Figure 13 shows the simulated and measured transmission coefficients of the system for both polarizations, with and without a 1 mm-wide crack on the bone tissue. In the case of the tangential incident E-field, the fractured area which is modeled by a blood layer, absorbs slightly more power due to the higher conductivity of blood. This causes a lower transmitted power to the receiver sensor in the fractured bone compared to the healthy bone, and the difference in received power increases as the crack width expands. In Fig. 13a, the transmission coefficient in a 1 mm-wide cracked bone is 4.4% less than that of a healthy bone, which is sufficient for image construction.

However, when the E-field is perpendicular to the crack, as shown in Fig. 13b, the transmission coefficient for the fractured bone is almost the same as that for a normal bone, indicating that EM waves do not sense any significant change in the bone tissue.

The utilization of lossy connectors and cables in experimental measurements could potentially account for the discrepancy between simulation and measurement results. Additionally, variations in the dimensions of different layers of the arm phantom and the amount of ingredients used in the fabrication process are other significant factors contributing to differences between simulation and measurement results. Fortunately, despite degradation in measured results, the detection mechanism is not affected as the system's performance basically relies on comparing normal and fractured bones for image construction, both of which are subject to the same fabrication/measurement errors. The normal bone serves as a calibration reference, and the variations in the received power of the sensor in the presence of a fracture are measured relative to it. In other words, the difference between the transmission coefficients of normal and fractured bones is of greater importance than the absolute values of the amplitude and phase of the transmission coefficients.

**Figure 14:**
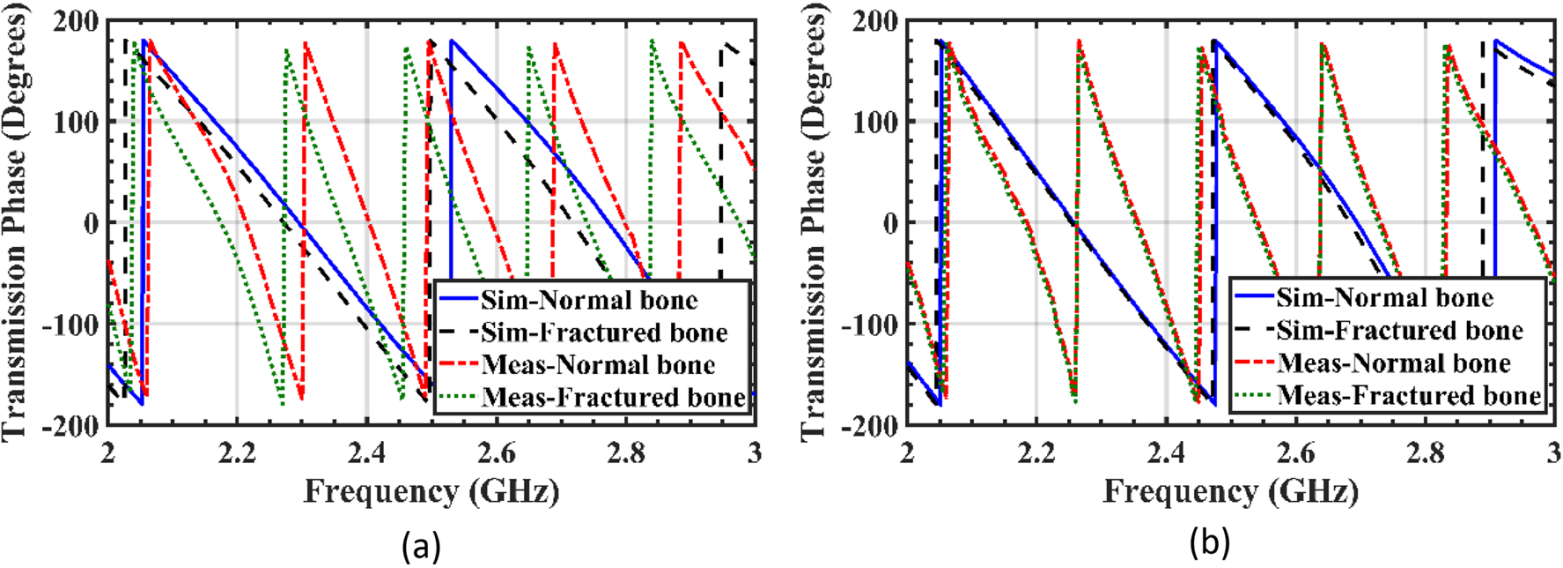
Comparison of the simulated and measured transmission phase of the sensor (∠S_21_) when placed on both the realized arm phantom and the simulated model with a 1 mm crack. The incident E-field is tested in two polarizations: (**a**) tangent to the crack orientation, (**b**) perpendicular to the crack orientation.

The phase analysis of transmission coefficient can also be carried out to detect the difference caused by the presence of cracks. Figure 14a and Fig. 14b show the phase of the transmission coefficient (∠S_21_) for the incident waves tangential and perpendicular to the crack orientation, respectively. The analysis of the transmitted signal phase shows that blood's higher permittivity results in greater phase relative to bone as the surrounding medium, as seen in Fig. 14a. Therefore, the significant difference between the phases of the transmitted signals in fractured and healthy bones suggests that utilizing phase differences could generate higher resolution images. However, the amplitude of transmission coefficients (|S_21_|) in Fig. 13 can also be used for bone imaging. The phase difference analysis provides valuable information about the location and extent of the fractures. As illustrated in Fig. 14b, similar to the amplitude analysis, when the incident E-field is perpendicular to the crack, the phase difference is negligible and the crack is not detected by the sensor.

**Figure 15:**
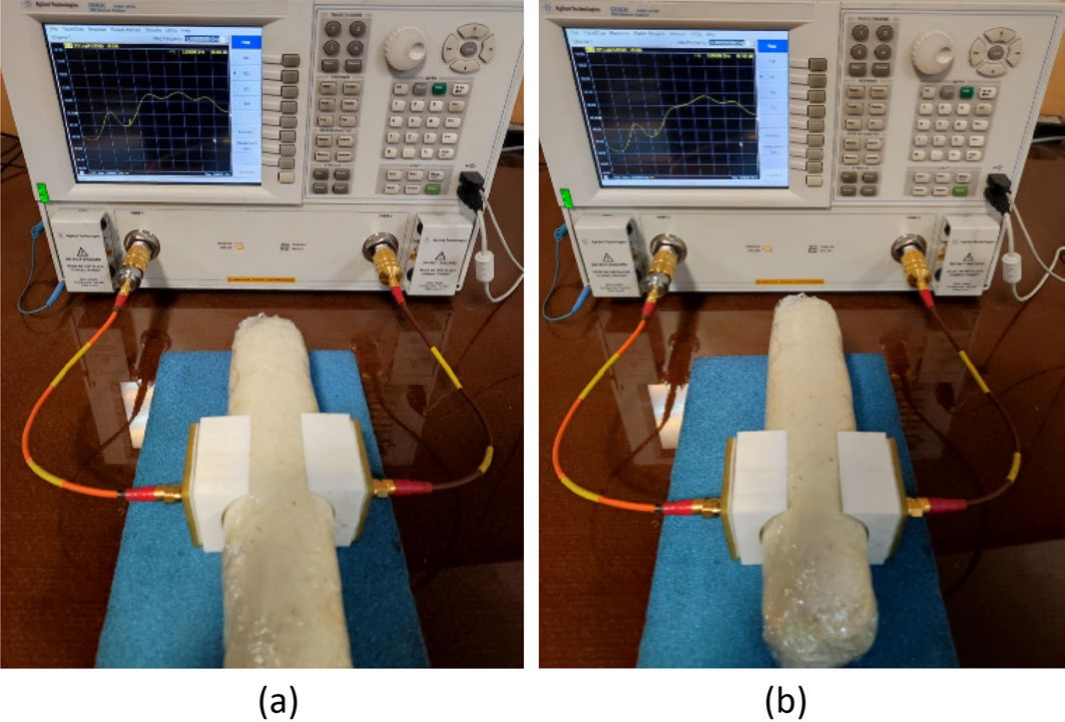
Experimental setup for raster scanning. (**a**) On the fractured region, (**b**) on the normal region.

To create an image of fractured bones, a raster scanning technique was utilized, as shown in Fig. 12. The incident waves were polarized tangentially to the crack's orientation during the scanning process. To simulate this process, longitudinal movement (*d*) was carried out in 9 steps from − 70 to 70 mm with respect to the crack location, which served as the scanning center (Fig. 12a). Additionally, rotation (*α*) around the body model was performed in 7 steps from − 30° to + 30°, as indicated in Fig. 12b. A total of 63 datasets were obtained and processed to construct the image of the cracked bone and determine the crack orientation. In actual measurements, a longitudinal scan was conducted in 5 steps from − 60 to 60 mm and a rotational scan was performed with 5 angles from − 40° to 40°, resulting in a total of 25 datasets that were used to create real images using a MATLAB image processing code. The code was used to establish an interface between CST Microwave Studio and MATLAB to enable the sampling of simulated S-parameters. Since the transmission phase (∠S_21_) exhibited noticeable variations due to the permittivity contrast between the blood layer in the fractured region and its surrounding bone, this parameter was used to construct bone images. The entire process was automated through the simulation of a MATLAB code, which executed CST software and sampled transmission phases at the desired frequency of 2.45 GHz. A two-dimensional matrix of transmission phase values was generated through raster scanning along both longitudinal and rotational axes, as shown in Fig. 12, and then linked and visualized as a 2D image of the damaged bone using a MATLAB function called “vis2D”^26^.The experimental setup illustrated in Fig. 15 allowed for the systematic scanning of the body tissue and the measured S-parameters were also imported into MATLAB, and the sampling technique was employed at the desired frequency.

In the first case, a 1 mm-wide transverse crack is created on the bone tissue in the center of the arm model, with the incident wave's polarization considered tangential to the larger dimension of the crack (y-polarized), as illustrated in Fig. 12a. In experimental measurements, the crack is modeled as a blood layer embedded in the phantom bone tissue. The phase of the transmission coefficient (∠S_21_) is measured at each step of the raster scan, and then is sampled at 2.45 GHz. The images are constructed from the difference between the phases of S_21_ when the sensors are positioned on the normal bone and when they are exactly on the fractured area.

**Figure 16:**
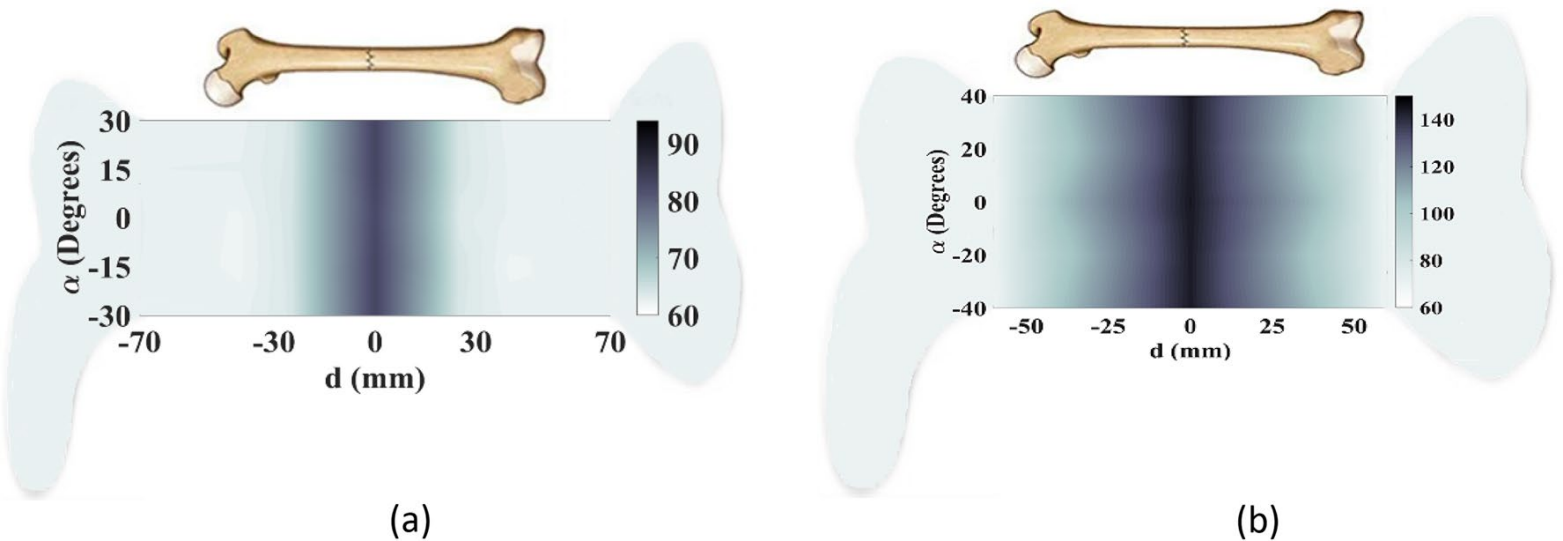
Constructed images of 1 mm-wide crack when the orientations of the incident E-filed and the crack are the same. (**a**) Simulated image, (**b**) measured image.

Simulated and measured images constructed by raster scanning are shown in Fig. 16, with color bars indicating the phase difference of the transmission coefficients. These images provide a visual representation of the crack in the bone tissue, with variations in phase indicating the presence and location of the crack. The comparison between simulated and measured images helps validate the performance of the proposed sensor system in detecting and localizing bone fractures.

**Figure 17:**
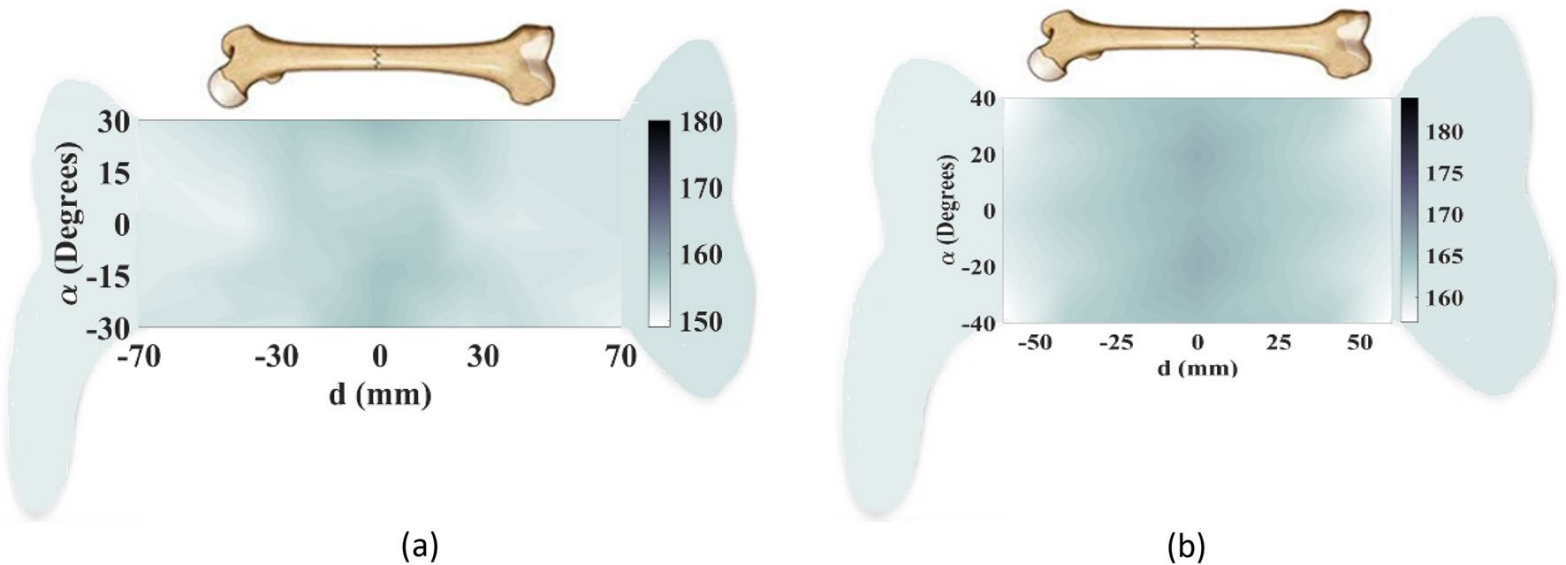
Constructed images of 1 mm-wide crack when the incident E-filed is perpendicular to the crack orientation. (**a**) Simulated image, (**b**) measured image.

Although the constructed images in Fig. 16 show the presence of the crack in the bone tissue, its orientation is not clear. To address this, the proposed sensor's dual-polarized capability is evaluated to recognize the crack's orientation. A raster scan is performed with an incident wave perpendicular to the crack (x-polarized), and the resulting images are shown in Fig. 17. Comparing the images obtained from excitation of two orthogonal polarizations, i.e. Figures 16 and 17, reveals that when the incident wave's polarization is tangential to the crack orientation, raster scanning around the damaged area provides useful information about the crack. However, when the incident wave is perpendicular to the crack, the resulting images do not contain any helpful information. Thus, to obtain useful data sets for constructing the image and detecting cracks, the incident E-field should be tangential to the crack orientation. The sensor's dual-polarized feature enables the detection and recognition of small cracks' orientation in bone tissue.

**Figure 18:**
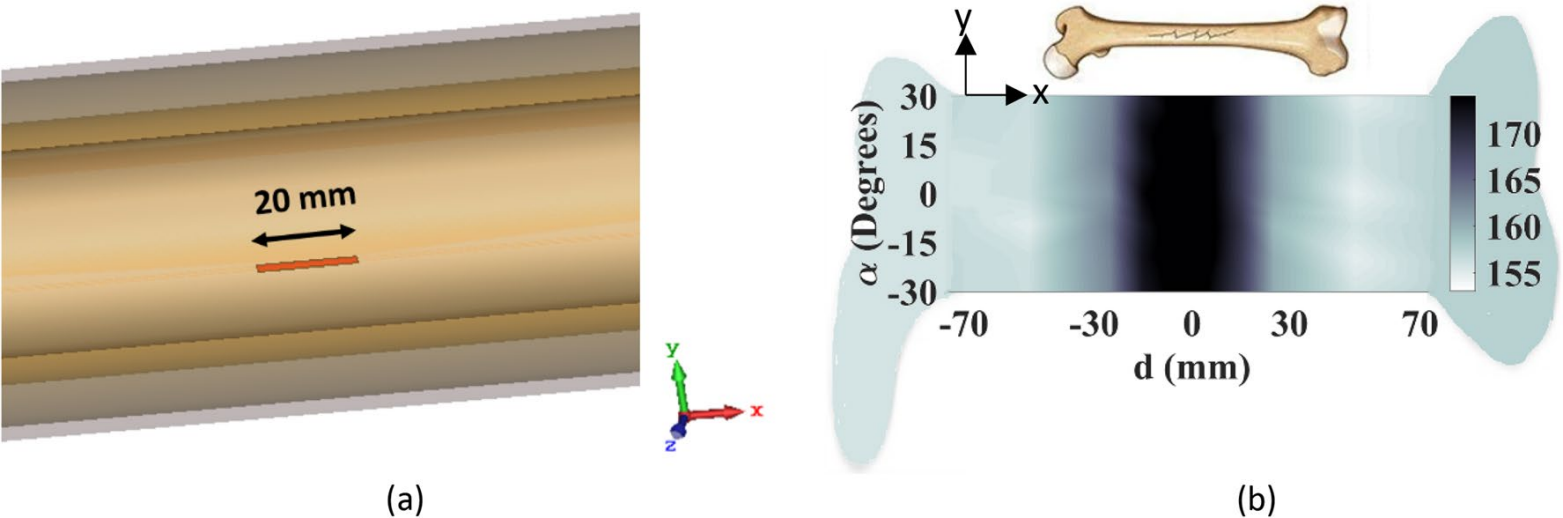
Longitudinal crack detection. (**a**) Crack orientation, (**b**) constructed image when the incident E-field is tangential to the crack.

The second scenario involves assessing the sensor's ability to detect a longitudinal 20 mm crack along the length of the bone, as depicted in Fig. 18a. By conducting a raster scan of the cracked bone using an incident wave that is tangential to the crack orientation (x-polarized), an image is generated, as illustrated in Fig. 18b. The resulting image clearly identifies the presence of the crack.

**Figure 19:**
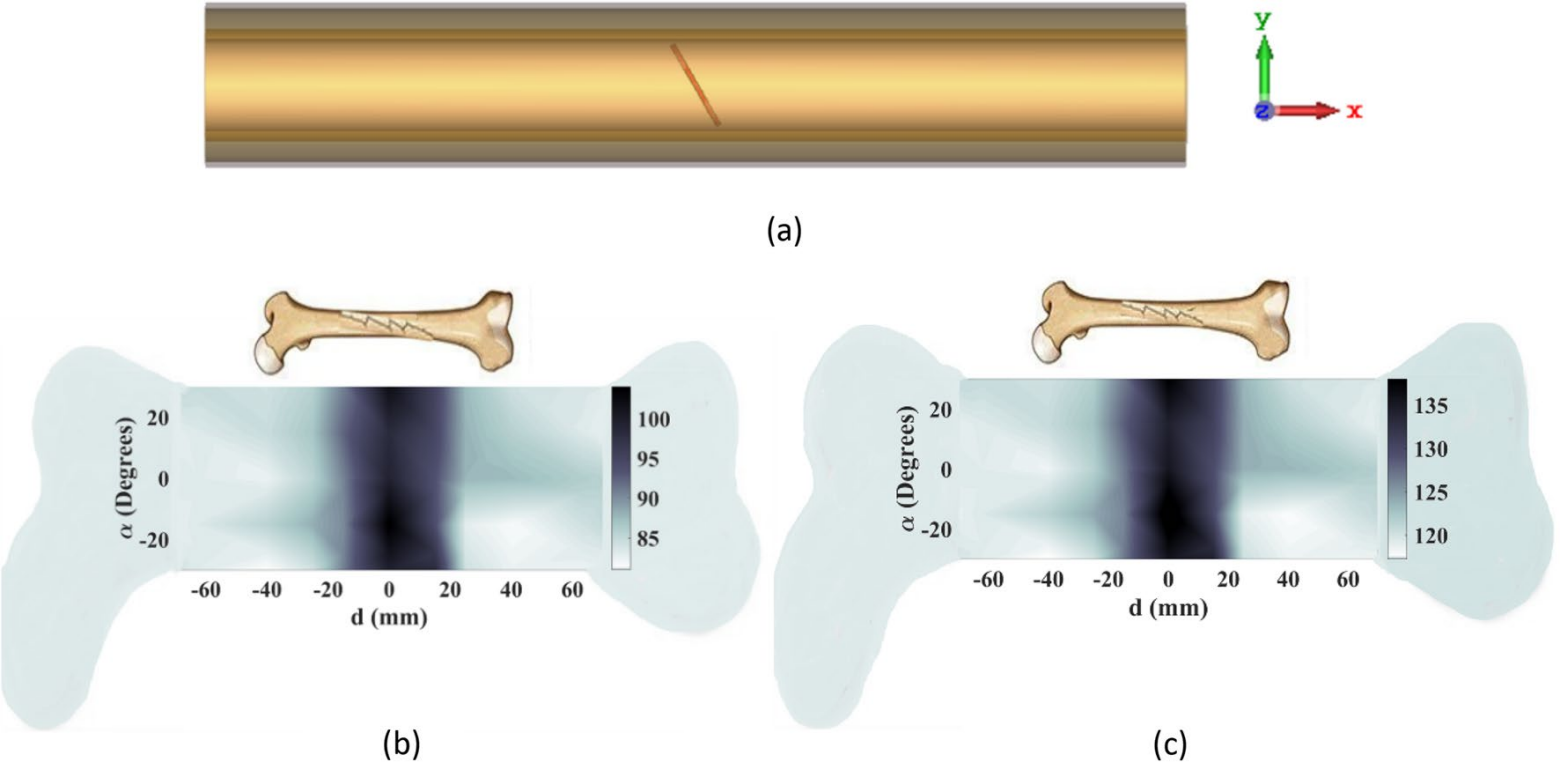
Evaluation of the transceiver system in detecting an inclined crack. (**a**) Crack orientation, (**b**) constructed image when the incident E-field is y-polarized, (**c**) constructed image when the incident E-field is x-polarized.

In order to evaluate the efficacy of the proposed transceiver sensor in detecting inclined fractures, a secondary simulation is conducted on a bone that exhibited such a fracture, as shown in Fig. 19a. It is predicted that in this case, the incident E-field in both polarizations can detect the inclined fracture. As demonstrated in Fig. 19b,c, when utilizing y-polarized and x-polarized E-fields, respectively, the constructed images of the affected bone reveal the presence of the fracture. These images provide clear evidence of accurate detection.

**Figure 20:**
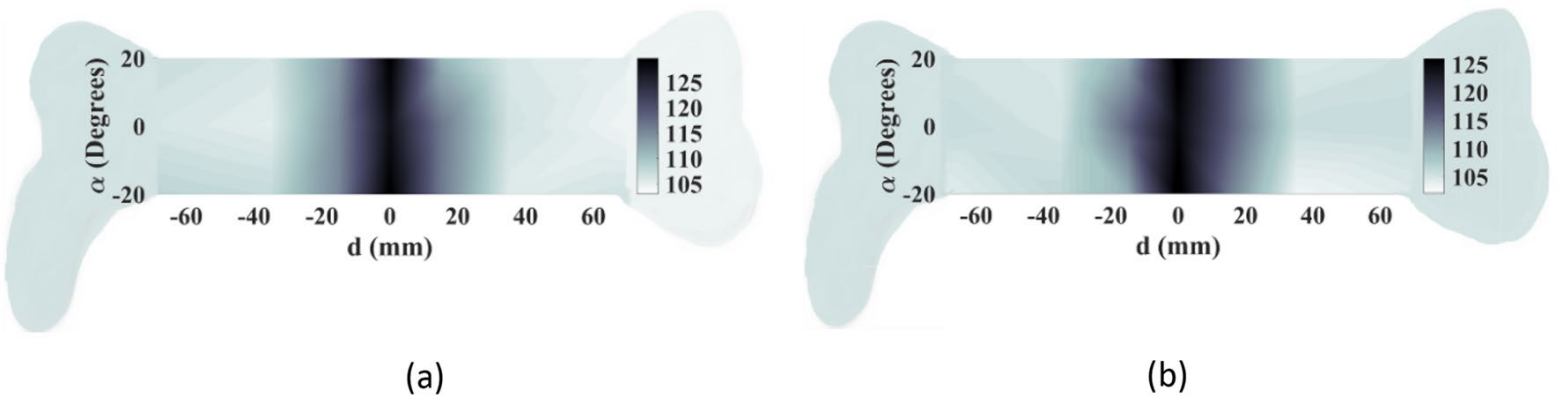
Evaluation of the sensitivity of the proposed sensor to various tissues and individual profiles. The image of the fractured area when (**a**) the skin layer is increased from 2 to 2.5 mm, (**b**) the fat layer is increased from 6 to 7 mm.

In order to evaluate the sensitivity of the proposed sensor to various parameters, such as variation of different body layers’ thicknesses, two additional image construction scenarios are carried out. The first scenario involved increasing the skin layer thickness from 2 to 2.5 mm, while the second scenario included thickening the fat layer from 6 to 7 mm, mimicking individuals with thicker fat layers. Raster scanning is employed, and images are achieved by calculating the difference in received power between normal and fractured bones. The resulting images, shown in Fig. 20a,b, respectively, demonstrate that the system can effectively adapt to various tissue characteristics and individual profiles, indicating its ability and reliability. These results illustrate that the performance of the system is not affected by differing people's profiles and tissues.

**Figure 21:**
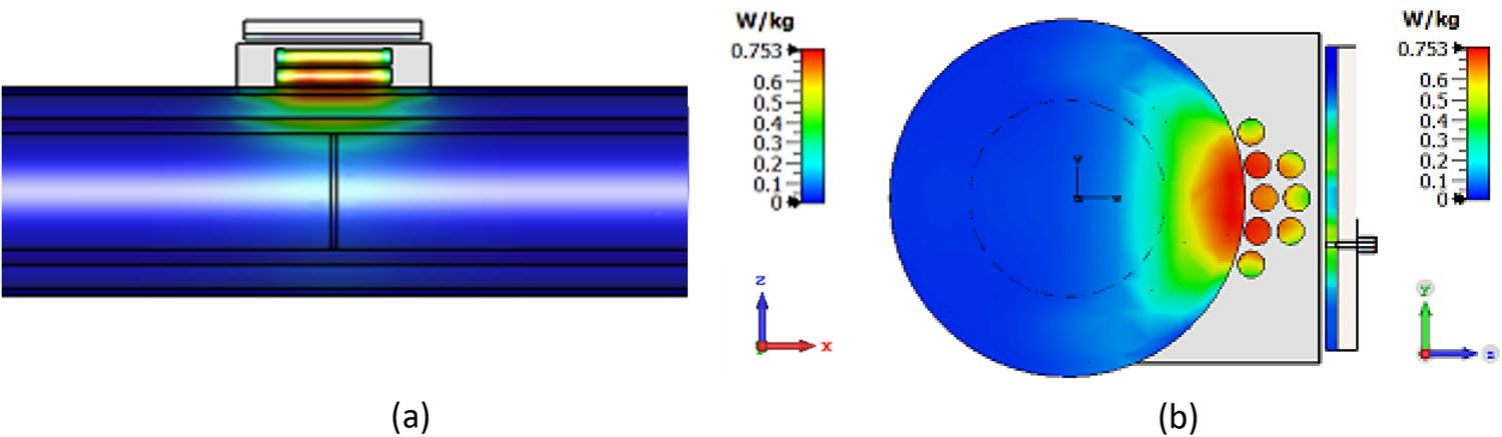
SAR analysis of the proposed sensor. (**a**) Top view, (**b**) side view.

The safe operation of microwave devices in biomedical applications is of paramount importance. To achieve this, an analysis of the Specific Absorption Rate (SAR) has been conducted to evaluate the safety of the proposed sensor. As shown in Fig. 21, the SAR distribution of the sensor when placed on a human body reveals a maximum SAR of 0.753 W/kg. This value is well below the standard maximum allowed limit of 1.6 W/kg, indicating that the proposed sensor operates safely and guarantees the individual's well-being during its use in biomedical applications.

Table 5 provides a comprehensive comparison of sensors used in bone damage detection systems. The analysis focuses on various parameters, such as dimensions, crack orientation, experimental measurements, and propagation analysis using matching mediums. It is worth noting that previous studies have primarily concentrated on detecting transverse cracks and have not considered evaluating crack orientation. However, the proposed crack detection system in this study presents several advantages in terms of miniaturization techniques, resulting in a smaller sensor size compared to other studies. Additionally, the use of a fabricated body phantom instead of dehydrated animal tissues is investigated to enhance the realism of the experimental setup.

**Table 5:** Literature review of the microwave bone damage detection systems.

References	Dimensions	Objective	Experimental measurements	Detection mechanism	Crack orientation	Matching medium	Detailed analysis
^5^	0.5λ × 0.66λ × 0.17λ	Bone health evaluation and osteoporosis detection	No measurement was carried out	Simulated S_11_ and S_21_	Transverse	No matching medium was introduced	- Lack of experimental measurements and propagation analysis - Single crack orientation detection
^7^	0.9λ × 0.65λ × 0.13λ	2 mm-wide bone crack detection	Tested on dehydrated porcine tissues	Evaluation of S_11_ and S_21_ and imaging algorithm	Transverse	No matching medium was introduced	- Bulky structure and - Impractical scanning method
^27^	comprised of 5 sets of transceivers, with horn antennas as the transmitter and Vivaldi antennas as the receiver with the overall diameter of 10λ	3 mm-wide bone lesion detection	Tested on liquid body phantom	Microwave tomography	Transverse	No matching medium was introduced	- Bulky and expensive structure - Lack of small lesions evaluation - Single crack orientation detection
^28^	0.9λ × 0.9λ × 0.03λ	1 mm-wide bone crack detection	Tested on dehydrated animal tissues	Evaluation of S_11_ of a single antenna in radar mode	Transverse	No matching medium was introduced	- Lack of wave concentration analysis - Single crack orientation detection - Bulky and large dimensions
The proposed sensor	0.36λ × 0.36λ × 0.037λ	1 mm-wide bone crack detection and localization	Tested on modified semi-solid realized arm phantom	Evaluation of S_11_ and S_21_ of the transceiver system, and data sampling for image construction	Transverse and longitudinal	Plano-concave modified lens structure	- Detailed propagation analysis - Evaluation of both transverse and longitudinal cracks - Miniaturized structure - Equipped with a novel matching lens

One notable feature of the proposed sensor is its ability to recognize both transverse and longitudinal crack orientations. This distinguishes it from other systems discussed in the literature. Moreover, the proposed sensor is highlighted for its reliability in precisely detecting narrow cracks that are at least 1 mm wide. Overall, the comparative analysis presented in Table 5 emphasizes the advantages of the proposed sensor in terms of miniaturization, crack orientation detection, reliability in narrow crack detection, and the use of a realistic body phantom.

## Conclusion

The proposed dual-polarized transceiver sensor is designed to detect and characterize narrow fractures in bone tissues. It comprises a square patch, a modified dielectric plano-concave lens, and a RIS layer at the bottom of the patch. The RIS layer introduces an additional inductive reactance, reducing the size of the sensor by 30% compared to conventional patch sensors and improving its accuracy in detecting narrow bone fractures. The novel inhomogeneous plano-concave lens as a superstrate layer, improves the concentration and penetration of EM power into damaged tissues by reducing wave divergence caused by the conductive nature of high-water content body tissues and the permittivity contrast between the patch sensor and body tissues. The lens also acts as a conformal adapting medium between the sensor and body curvature. It contains via holes filled with a lossy dielectric material similar to human fat tissue, to concentrate EM power around the patch, promoting normal propagation to body tissues with minimal deviation. The sensor's capabilities were demonstrated through raster scanning of a human arm using two identical sensors, as a transmitter and receiver, placed opposite each other on the tissue. A cylindrical semi-solid gelatin-based arm phantom was utilized to simulate the behavior of a human arm during measurements. By measuring the transmitted power between them through the body, images of the damaged tissue were constructed to recognize the location and orientation of cracks in the mm range.

Images were constructed by analyzing the scattering parameters in the frequency domain, particularly the transmission phase (∠S_21_), which exhibited clear discrepancy between the normal and fractured bones. An image processing algorithm was used to extract meaningful and clear images by sampling data at arbitrary frequencies within the bandwidth of the sensor. The dual-polarized operation of the sensor allows for the recognition of the crack orientation. Tangential E-field to the crack orientation proves to be more effective in crack detection, while perpendicular E-field does not provide significant information for bone imaging. Various cases of vertical, longitudinal, and inclined cracks were considered to validate the system's reliability. The sensor's sensitivity to varying tissue thicknesses was investigated, and specific absorption rate (SAR) analyses were conducted to ensure the immune performance of the sensor. The adaptation to the curvature of the body and optimal performance on human body in the detection and localization of narrow bone fractures are prominent features of this novel sensor system, making it a promising solution in the field of bone imaging.

## Data Availability

The datasets generated during and/or analyzed during the current study are available from the authors on reasonable request.
